# Perioperative Ultrasound-Guided Continuous Caudal Epidural Analgesia in Newborns: A Case Series in a Tertiary Medical Center

**DOI:** 10.7759/cureus.48272

**Published:** 2023-11-04

**Authors:** Filipa Portela, Gabriela Costa, Teresa Cenicante

**Affiliations:** 1 Anesthesiology, Centro Hospitalar Lisboa Ocidental, Lisbon, PRT; 2 Anesthesiology, Hospital Dona Estefânia, Lisbon, PRT

**Keywords:** neonatal care, caudal analgesia, neonatal intensive care unit (nicu), caudal epidural, general pediatric surgery

## Abstract

Background

Caudal epidural anesthesia technique is a relevant method for postoperative analgesia in newborns, allowing for the reduction of drug-induced respiratory depression. The threading of a catheter is, however, uncommon in clinical practice. Our main purpose was to describe our experience regarding caudally inserted epidural catheters in neonates undergoing major abdominal surgery.

Methods

We included every full-term neonate undergoing surgery under combined caudal epidural-general anesthesia from 2017 to 2022 in our institution. After induction of general anesthesia, an ultrasound-guided caudal epidural injection was performed, and an epidural catheter was inserted for perioperative analgesia. An epidural bolus of ropivacaine was administered to every patient before the surgical incision, and an epidural infusion of ropivacaine 0.05% was administered for 24 hours.

Results

Retrospectively obtained data included six full-term neonates with American Society of Anesthesiologists (ASA) physical status II to IV. Intraoperatively, good analgesia was achieved without hemodynamic instability or need for additional systemic opioids after induction. At the end of surgery, five of the six neonates were extubated without adverse respiratory events. Postoperatively, effective analgesia was achieved in four cases with an epidural infusion of ropivacaine 0.05%, at a rate between 0.2 and 0.4 mg/kg/h, and intravenous paracetamol. Epidural pain control was not successful in one neonate, and thus an intravenous fentanyl infusion was added. The sixth neonate remained intubated for prolonged mechanical ventilation due to surgical complications, and thus an intravenous fentanyl infusion was introduced for sedation in the neonatal intensive care unit (NICU), not allowing to evaluate the effectiveness of the epidural infusion alone. No other complications related to the epidural catheters were reported.

Conclusion

Continuous caudal epidural analgesia may be a valuable technique with a low risk of complications, decreasing the incidence of respiratory adverse events in this patient population. Although more cases are needed for a stronger conclusion, it has become a useful analgesic strategy for major abdominal surgery in neonates in our institution.

## Introduction

Nociceptive pathways in neonates are not a lessened version of adult ones, with neuroanatomical and neurophysiological components being sufficiently developed to allow for pain transmission [1-2]. In this regard, reducing exposure to noxious stimuli during surgical stress in this patient population is as important as in adults in order to avoid adverse physiological and psychological sequelae such as chronic pain [2-3].

However, the increased risk of perioperative respiratory depression is a feature of premature infants and newborns that poses difficulties regarding analgesia [4]. In reality, there might be some reluctance to administer adequate doses of systemic opioids due to the risk of delayed tracheal extubation in this age group [5]. In this setting, caudal anesthesia has gained significant interest since its introduction by Meredith Campbell in 1933, allowing for a reduction of drug-induced respiratory depression and a more rapid resumption of spontaneous breathing [4-6].

Regional anesthesia techniques might lead to a reduction in the development of persistent postsurgical pain, offering long-term advantages. The underlying mechanism is not yet fully explained, but the prevention of painful stimulus from reaching the central nervous system and the decrease in the total amount of general anesthetics administered seem to play an important role [7]. Caudal blocks, by avoiding the short skin-epidural distance of the lumbar and thoracic epidurals, may be even more advantageous [4].

Although the term “caudal block” is mainly used for single injection procedures in the pediatric setting, a caudal approach can also be used for threading an epidural catheter. Nonetheless, this variant is uncommon in clinical practice, constituting only 1% of cases in a follow-up one-year prospective survey of the French-Language Society of Paediatric Anaesthesiologists (ADARPEF) [8].

The main purpose of our study is to describe our experience regarding caudally inserted epidural catheters in neonates undergoing major abdominal surgery.

## Materials and methods

After approval by the hospital ethics committee and obtaining informed parental consent, we retrospectively included every full-term neonate (children under 28 days of age with a total gestation period of at least 37 weeks and less than 42 weeks) who underwent surgery under a combined caudal epidural-general anesthesia technique in the last six years (2017-2022) at Hospital Dona Estefânia, Lisbon, Portugal.

Demographic features, surgical procedure, description of the technique, perioperative analgesics, and postoperative clinical course were recorded.

Anesthetic technique

Preoperatively, internationally recommended fasting times were assured, and no premedication was administered. Monitoring included pulse oximetry, electrocardiography, noninvasive arterial blood pressure, capnography, and core temperature.

General anesthesia was induced intravenously with propofol or with inhaled sevoflurane whenever no intravenous catheter was present. A 1-2 µg/kg fentanyl bolus was administered in every case followed by 3-5 mg/kg of propofol. Neuromuscular blockade was achieved with rocuronium 0.6-1 mg/kg in four patients and cisatracurium 0.15 mg/kg in one patient. In all cases, the trachea was intubated using direct or video-assisted laryngoscopy, and general anesthesia was maintained with sevoﬂurane in oxygen/air.

**Figure 1:**
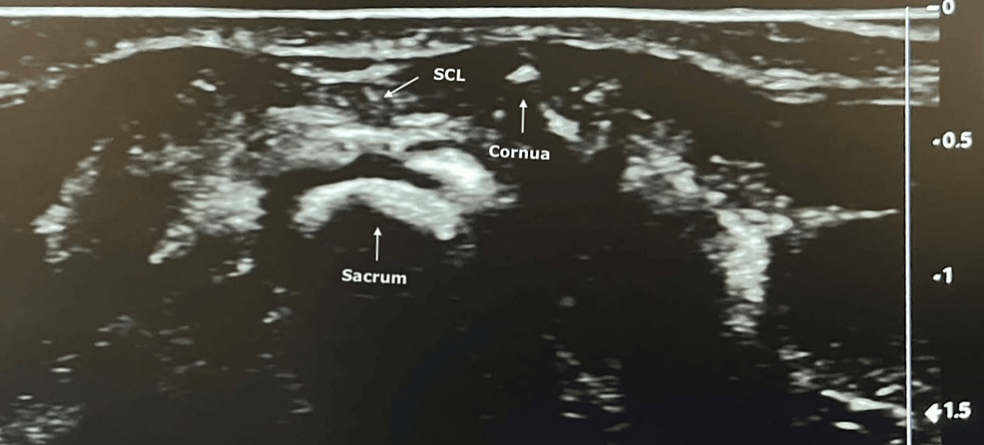
Ultrasound view of the transverse plane SCL, sacrococcygeal ligament

The neonates were positioned in lateral decubitus with hips flexed, and the skin was desinfected with Octiset®, an aqueous antiseptic solution. Before starting the technique, the catheter’s insertion length was visually estimated in accordance with the dermatomes predictably affected by the surgical incision. The two sacral cornua were identified by palpation, and a linear ultrasound probe was placed in the transverse plane across them. The sacrococcygeal ligament and the deeper sacral bone were visualized as hyperechoic structures (Figure 1).

**Figure 2:**
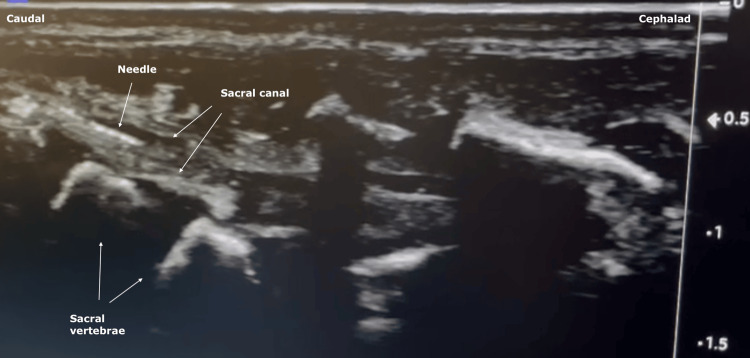
Ultrasound view of the longitudinal plane

A 20G dull Crawford-type bevel Epican™ needle was inserted into the space between the sacrococcygeal ligament and the sacral bone and in the middle of the two cornua, with the bevel pointing cephalad. The transducer was then turned by 90º to a longitudinal view to insert the needle in-plane into the sacral hiatus (Figure 2).

**Figure 3:**
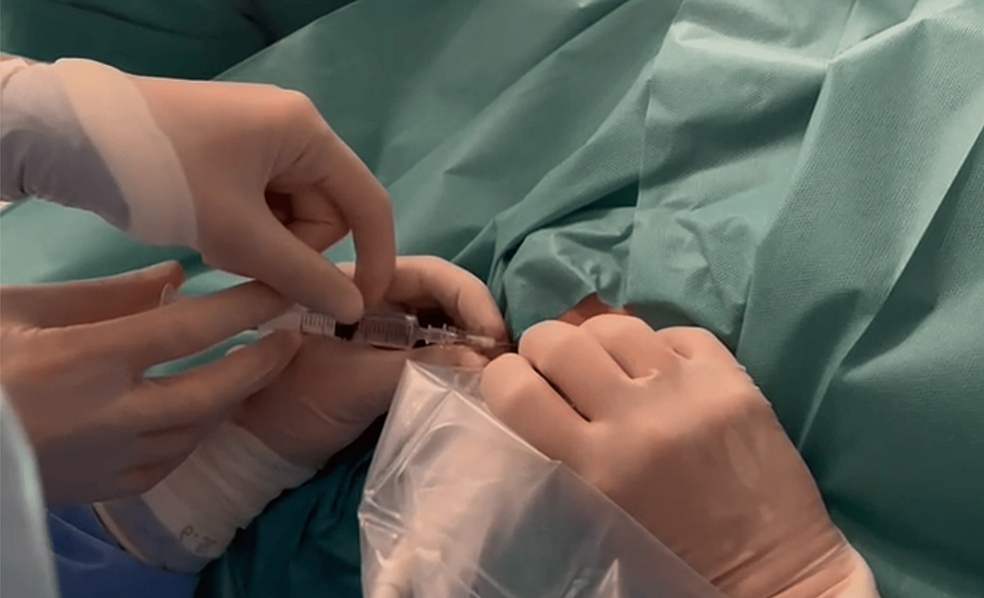
Caudal epidural four-handed technique

Afterward, an epidural bolus of 2 mL normal saline solution was administered to facilitate catheter progression. An epidural Perifix® ONE 24G catheter (B. Braun, Melsungen, Germany) was posteriorly threaded into the low thoracic epidural space, up to a level according to the surgical incision. This was done under ultrasound guidance with the help of a second anesthetist, with a four-handed technique (Figure 3).

The exact location of the tip of the catheter was always visualized and registered in most cases. An epidural bolus of ropivacaine 0.1% to 0.2% was administered in accordance with Takasaki’s formula (0.056 x kg x number of segments) [9], and the insertion site was covered with an occlusive waterproof dressing.

Intraoperative course

Throughout the surgical procedure, further epidural boluses were administered as needed. During the procedure, every intervention and medication administered were registered in the anesthesia record. After the end of surgery, in most cases, the trachea was extubated in the operating room, and the neonates were transferred to the neonatal intensive care unit (NICU).

Postoperative management

In the postoperative period, an epidural infusion of ropivacaine 0.05% was administered in a range of 0.2 to 0.4 mg/kg/h, which was managed by an anesthetist in the intensive care unit (ICU). Pain was regularly assessed with five behavioral indicators in conformity with the Neonatal Pain, Agitation, and Sedation Scale (N-PASS) with a possible score of 0 to 10 [10], and the epidural infusion was adjusted accordingly. In this regard, if N-PASS score was 0-2, the epidural infusion was maintained, and if it was higher, it was increased until 0.4 mg/kg/h. The catheter was removed by the on-call anesthetist 24 hours after the surgery or earlier in case of complications, such as leakage around it.

## Results

Retrospectively obtained data included six full-term neonates with American Society of Anesthesiologists (ASA) physical status II to IV [11] from January 2017 to December 2022. These patients underwent surgical procedures performed via an abdominal incision, which included colostomy, ileostomy, open Ladd’s procedure, Meckel’s diverticulectomy, omphalocele repair, and intestinal resection.

The sacral epidural space was easily accessed with one or two attempts in all infants without difﬁculty advancing the epidural catheter. In all cases, the correct catheter placement was confirmed by ultrasound to be at T8-T12. There was no evidence of inadvertent intravascular or intrathecal injection of local anesthetic or misplacement of any catheter.

The following tables describe the perioperative details of each case, focusing on patient demographics, preoperative diagnosis, and surgery performed (Table 1), and caudal epidural analgesia and postoperative pain (Table 2).

**Table 1:** Patient demographics, preoperative diagnosis, and surgery performed ASA, American Society of Anesthesiologists; F, female; M, male

Patient	Postnatal age	Gestational age at birth (weeks + days)	Sex	ASA	Weight (kg)	Diagnosis	Surgery
1	4 days	39+6	M	II	3.1	Intestinal malrotation	Open Ladd’s procedure
2	<24 hours	39+3	F	II	3	Meckel’s diverticulum and minor omphalocele	Meckel’s diverticulectomy and omphalocele repair
3	1 day	41+1	M	III	3.1	Anorectal atresia	Colostomy
4	2 days	39+1	F	II	3.4	Ileal atresia	Ileostomy
5	20 days	38+6	M	II	2.8	Intestinal malrotation	Open Ladd’s procedure
6	17 days	40+2	M	IV	3.8	Intestinal volvulus	Intestinal resection surgery

**Table 2:** Details of caudal epidural analgesia and postoperative pain N-PASS, Neonatal Pain, Agitation, and Sedation Scale; T, thoracic vertebrae

Patient	Level of catheter/length of insertion	Intraoperative epidural bolus	Postoperative epidural infusion	Tracheal extubation	Catheter removal	N-PASS score range during 24 hours	Caudal catheter complications
1	Not registered	Ropivacaine 0.2% 3 mL	Ropivacaine 0.05% 1 mL/h	End of surgery	24h	0-6	Insufficient analgesia
2	Not registered	Ropivacaine 0.1% 2 mL	Ropivacaine 0.05% 1 mL/h	End of surgery	24h	0	No
3	T11-T12/9 cm	Ropivacaine 0.2% 1 mL	Ropivacaine 0.05% 1.2 mL/h	End of surgery	24h	0	No
4	13 cm	Ropivacaine 0.1% 3.5 + 2 mL	Ropivacaine 0.05% 2 mL/h	End of surgery	24h	1-2	No
5	T10	Ropivacaine 0.1% 1 + 1 mL	Ropivacaine 0.05% 1 mL/h	End of surgery	24h	0-1	No
6	12 cm	Ropivacaine 0.1% 1.5 mL	Ropivacaine 0.05% 2 mL/h	5 days after the first surgery	20h	1-2	Leakage around catheter insertion

Intraoperative course

Hemodynamic stability was achieved in all patients, and there was no need for additional intravenous opioid boluses throughout the surgery. In longer cases (patients 4 and 5), there was a second administration of epidural ropivacaine 2 hours after the beginning of surgery. No technical problems, such as disconnections, catheter kinking, and blockages, were recorded, and there were no signs of adverse systemic toxicity intraoperatively.

Postoperative respiratory outcome

Five neonates were extubated at the end of surgery and remained eupneic in the NICU with no need for supplemental oxygen. No respiratory adverse events were registered postoperatively in these patients. One high-risk infant (patient 6), who was diagnosed with intestinal volvulus and undergoing an intestinal resection, was not extubated immediately after surgery due to surgical complications and a very high probability of need for a reoperation. Mechanical ventilation was continued in the NICU, and this neonate underwent a relaparotomy and further intestinal resection three days later.

Postoperative analgesia

Epidural pain management was successful in four cases (patients 2, 3, 4, and 5), managed with an epidural infusion of ropivacaine 0.05% at a rate of 0.2 mg/kg/h and paracetamol as the sole other analgesic. In these patients, there was no need to readjust the epidural infusion rate. The N-PASS score for the first 24 hours was 0 in patients 2 and 3, 1-2 in patient 4, and 0-1 in patient 5. These newborns remained awake without signs of discomfort, and the on-call consultant anesthetist removed the catheter 24 hours postoperatively.

Patient 1 presented uncontrolled pain one hour and a half after the beginning of the epidural infusion with an N-PASS score of 6. By this time, an epidural bolus of 2 mL of ropivacaine 0.05% was administered by the on-call anesthetist with no success (N-PASS score of 6). One hour later, an intravenous fentanyl infusion was started with effective pain control (N-PASS score of 0). Subsequently, in the first 24 hours postoperatively, an epidural infusion was maintained at a rate of 0.2 mg/kg/h together with the intravenous fentanyl infusion, with an N-PASS score of 0-1.

Patient 6 remained intubated in the postoperative period, and, therefore, an intravenous fentanyl infusion was started right after ICU admission. Together with an epidural infusion, this led to effective pain control (N-PASS score of 0-1). However, leakage around the epidural catheter was detected at 20 hours postoperatively, and the on-call consultant anesthetist decided to remove the catheter earlier. Afterward, postoperative analgesia was accomplished solely with the intravenous fentanyl infusion and paracetamol.

On removal of the catheter, no signs of inflammation of the skin were visualized, and the tip of the catheter was intact in all cases. Moreover, no infectious complications related to the epidural catheter or signs of adverse systemic toxicity were reported in the postoperative period.

## Discussion

The aim of our study was to describe our experience regarding the perioperative use and advantages of ultrasound-guided caudally inserted epidural catheters in neonates. In this age group, epidural analgesia has gained popularity in the perioperative period, allowing for a reduction of opioid-related adverse effects and minimizing respiratory depression [4]. It has also granted a timely recovery of spontaneous ventilation and earlier tracheal extubation [5].

Enhanced Recovery After Surgery (ERAS®)

In 2020, the Enhanced Recovery After Surgery (ERAS®) Society published the Consensus Guidelines for Perioperative Care in Neonatal Intestinal Surgery, which focus on an opioid-sparing multimodal analgesia strategy for perioperative neonatal management [12]. In this patient population, morphine has some specific disadvantages such as a narrow therapeutic window with a higher risk of respiratory depression, hypotension, and decreased gastrointestinal motility. In this regard, regional anesthesia may reduce the need for perioperative opioids, and epidural analgesia decreases respiratory complications and shortens the time to bowel function.

Caudal versus lumbar/thoracic approach

Regarding abdominal surgery in neonates, an epidural catheter issues excellent intra- and postoperative analgesia, tackling parietal and visceral pain [13]. These catheters can be inserted directly at the lumbar or thoracic spine or, as is practiced in our institution, caudally.

In fact, the ease of caudal catheter insertion in neonates, due to the absence of adult-like vertebral curvature and softness of epidural fat, has been described as reasoning for preferring this technique [14]. Although the literature still shows no robust evidence on the safest route of insertion, it has been suggested that the less experienced anesthetist should consider the caudally inserted epidural catheter [14-15]. This technique is easy to learn in the pediatric setting, avoiding the short skin-epidural distance of the lumbar and thoracic epidurals and the consequent higher risk of dural puncture [4].

Landmark-based versus ultrasound-guided technique

Landmark-based palpation of the sacral hiatus cannot guarantee a correct technique, and its known risks include intravascular access and dural puncture [4].

The superiority of ultrasound-guided puncture seems obvious, although there is little evidence associating ultrasound-guided caudal epidural anesthesia with reduced morbidity or improved success rates. In fact, periprocedural ultrasound guidance is still the exception and was only used in 11% of caudal-to-thoracic epidural catheters in pediatric patients in a multi-institutional study of the Paedriatic Regional Anesthesia Network (PRAN) [16]. However, it was recently reported that the sacral hiatus is clinically identiﬁable only if the sacral cornua are palpable, with ultrasound being essential in all other cases [17].

In our institution, ultrasound-guided regional anesthesia is the gold standard for peripheral techniques and also a common practice for caudal catheter insertion in neonates.

Analgesia efficacy

In our NICU, postoperative pain is evaluated with N-PASS. This score has been developed for the purpose of assessing pain, agitation, and sedation levels in critically ill infants, and has been considered a valid and reliable tool in ventilated and/or postoperative newborns [10].

In our study, an epidural infusion was the main postoperative analgesic approach in four patients, with paracetamol as the sole other analgesic administered. These newborns had N-PASS scores of 2 or less in the first 24 hours, with two of them remaining at 0 for this whole period.

Patient 1 presented with uncontrolled pain one hour and a half after the beginning of the epidural infusion, which resolved only after an intravenous fentanyl infusion was added. Since the patient’s catheter level and insertion length were not registered, we speculate that the patient’s epidural and incision level may not have matched, perhaps leaving some dermatomes untargeted that were necessary for effective analgesia. Other factors that may explain insufficient analgesia are unilateral block (inadequate epidural solution spread or catheter migration towards one side) and epidural failure (catheter was not in epidural place) [18].

In patient 6, an intravenous fentanyl infusion was added right after admission to the NICU for sedation for tracheal intubation and mechanical ventilation. As a result, we could not assess the analgesic efficacy of the epidural infusion alone.

Complications and safety

According to a large European multicenter study by ADARPEF, regional anesthesia in children is remarkably safe, with a complication rate of only 0.12%, and the use of catheters does not seem to alter this safety. In this study, out of 8,493 caudal epidurals, only eight patients with complications were identified: six dural taps (with no postdural puncture headache), one nerve injury, and one case of cardiac toxicity. In fact, the main problems associated with caudal epidurals seem to be periprocedural, related to difficulty in placing the catheter, and the most common adverse event was an abandoned block [8,16].

In our study, we report no safety issues and two postoperative complications: leakage around the catheter (patient 6) and insufficient analgesia (patient 1). Furthermore, in our institution, we use occlusive waterproof dressings and remove epidural catheters in neonates after 24 hours to prevent infectious complications and have had none to report.

## Conclusions

A continuous caudal epidural technique seems to be a valuable method for perioperative analgesia in newborns, providing adequate pain management while avoiding the risk of a lumbar/thoracic approach in this age group. We reported six cases, of which four were totally successful, which leads us to conclude that more research is needed. However, our colleagues in the NICU have encouraged us to continue performing this technique as it has had a positive outcome in our institution, becoming a relevant strategy for the perioperative management of neonates undergoing major abdominal surgery.
